# Prescribing Metformin to Prevent Antipsychotic-Induced Weight Gain – a Clinical Audit and Clinician Stakeholder Event

**DOI:** 10.1192/bjo.2026.11718

**Published:** 2026-06-30

**Authors:** Sarah Fynes-Clinton, Piyumi Dona, Zoe Scabbiolo, Jakov Zlodre, Monty Lyman

**Affiliations:** Oxford Health Foundation Trust, Oxford, United Kingdom

## Abstract

**Aims:**

Antipsychotic-induced weight gain (AIWG) is common, contributes substantially to cardiometabolic morbidity, and is a major cause of treatment discontinuation. Recent international consensus guidelines recommend metformin for the prevention of AIWG in high-risk patients. This project examined whether eligible inpatients were being identified, whether metformin was being discussed, and whether it was being initiated in line with these recommendations.

**Methods:**

A retrospective audit (April-June 2025) was conducted on an acute psychiatric ward to identify patients meeting criteria for pharmacological prevention of AIWG. Following an education-based intervention for clinicians, a re-audit was completed (June-September 2025). Data were extracted from electronic health records. A clinician questionnaire was administered at a stakeholder event involving psychiatry and GP trainees (n=18) to explore barriers and facilitators to preventative metformin prescribing.

**Results:**

At baseline, 60% (27/48) of patients met criteria for preventative metformin, yet only one patient had a documented discussion and one was initiated on treatment. Following the intervention, 64% (32/57) met criteria. Of those that met criteria, documented discussions increased to 34% (11/32), and initiation rose to 9% (3/32). Among clinicians surveyed, 94% (16/17) reported not routinely prescribing metformin preventatively. Key barriers included absence of local guidelines (78%), concerns about polypharmacy (56%), and perceived reluctance in primary care to continue prescribing (33%).

**Conclusion:**

Despite clear eligibility and international guidance, preventative metformin prescribing for AIWG remained uncommon, with education alone producing only modest improvements. Barriers appear primarily structural and inter-professional rather than evidence based. Development of local guidelines and improved psychiatrist-GP communication may be necessary to translate international recommendations into routine clinical practice.

